# The Effects of Lycopene on the Methylation of the GSTP1 Promoter and Global Methylation in Prostatic Cancer Cell Lines PC3 and LNCaP

**DOI:** 10.1155/2014/620165

**Published:** 2014-10-20

**Authors:** Li-Juan Fu, Yu-Bin Ding, Lan-Xiang Wu, Chun-Jie Wen, Qiang Qu, Xue Zhang, Hong-Hao Zhou

**Affiliations:** ^1^Institute of Life Sciences, Chongqing Medical University, No. 1 Yixueyuan Road, Chongqing 400016, China; ^2^Traditional Chinese Medicine, Chongqing Medical University, Chongqing 400016, China; ^3^Pharmacogenetics Research Institute, Central South University, Changsha, Hunan 410078, China

## Abstract

DNA (cytosine-5-) methylation silencing of GSTP1 function occurs in prostate adenocarcinoma (PCa). Previous studies have shown that there is an inverse relationship between dietary lycopene intake and the risk of PCa. However, it is unknown whether lycopene reactivates the tumor suppressor gene glutathioneS-transferase-*π* (GSTP1) by demethylation of the hypermethylated CpGs that act to silence the GSTP1 promoter. Here, we demonstrated that lycopene treatment significantly decreased the methylation levels of the GSTP1 promoter and increased the mRNA and protein levels of GSTP1 in an androgen-independent PC-3 cell line. In contrast, lycopene treatment did not demethylate the GSTP1 promoter or increase GSTP1 expression in the androgen-dependent LNCaP cell line. DNA methyltransferase (DNMT) 3A protein levels were downregulated in PC-3 cells following lycopene treatment; however, DNMT1 and DNMT3B levels were unchanged. Furthermore, the long interspersed element (LINE-1) and short interspersed element ALU were not demethylated when treated by lycopene. In LNCaP cells, lycopene treatment did not affect any detected DNMT protein expression, and the methylation levels of LINE-1 and ALU were decreased. These results indicated that the protective effect of lycopene on the prostate is different between androgen-dependent and androgen-independent derived PCa cells. Further, in vivo studies should be conducted to confirm these promising results and to evaluate the potential role of lycopene in the protection of the prostate.

## 1. Introduction

Prostate cancer (PCa) is the most common nonskin malignancy and a leading cause of cancer-related mortality in men of Western countries [1]. The earliest somatic genome change that occurs in PCa in humans is the* de novo* DNA methylation of glutathione* S*-transferase-*π* (GSTP1) accompanied by epigenetic gene silencing [2]. This silencing, mediated by hypermethylation of GSTP1 transcriptional regulatory sequences, has been consistently detected in more than 90% of PCa cases [3, 4], and this specificity of GSTP1 promoter methylation measured in body fluids (plasma, serum, and urine) suggests a potential screening measurement for prostate cancer diagnosis [5]. Loss of GSTP1 function may contribute to the early stages of PCa development, with GSTP1 methylation evident in 5–10% of proliferative inflammatory atrophy (PIA) precursor lesions, and 70% of high-grade prostatic intraepithelial neoplasia (PIN) lesions, respectively [6]. In contrast, hypermethylation of GSTP1 is rarely detected in benign prostatic epithelium and patients without disease [7].

GSTP1 is a detoxifying enzyme that acts in a multifunctional manner to protect cells from genome-damaging stresses caused by electrophiles and oxidants. GSTP1 silencing, and a resultant loss of enzymatic protection against reactive chemical species, may provide an explanation for the well-known sensitivity of human prostatic carcinogenesis to environmental and lifestyle influences [8, 9]. For example, mice with disrupted Gstp1/2 genes are more susceptible to developing skin tumors when exposed to topical carcinogenic chemicals [10].

Unlike genetic mutations, DNA methylation does not change the DNA sequence itself. Methylation is a potentially reversible modification, which makes it amenable to therapeutic intervention [11]. This has important implications for chemoprevention because, as mentioned above, altered GSTP1 activity and expression are largely due to GSTP1 DNA hypermethylation in the promoter 5′ in more than 90% of PCA cases [3, 4].

Dietary compounds may prevent cancer through DNA methylation modifications in the cells [12]. Indeed, some investigators have investigated the effects of (2)-epigallocatechin-3-gallate (EGCG) [13], curcumin [14], and catechins on DNA methylation in different types of cells. Studies showed that green tea polyphenols are excellent candidates for the chemoprevention of prostate cancer reexpression of GSTP1 in human prostate cancer cells [15], as they induce GSTP1 expression through demethylation of its promoter in human prostate cancer cells.

Lycopene, a member of the carotenoid family, is naturally abundant in tomatoes [16]. As humans do not synthesize lycopene and half of the carotenoids in the human serum are provided by dietary consumption of lycopene, an adequate consumption of nutrients is critical [17]. Lycopene is a potential antioxidant or nutrient protector. A number of epidemiological studies have shown an inverse relationship between dietary lycopene intake and the risk of PCa [18, 19]. Clinical intervention studies have found that short courses of tomato or lycopene supplementation in men with PCa decreased the carcinogenesis of advanced PCa, reduced DNA damage, increased apoptosis of cancer cells, and decreased serum prostate-specific antigen concentrations [20, 21].

However, the ability of lycopene to affect epigenetic changes has this far only been examined in one study in which lycopene was shown to demethylate the promoter of the GSTP1 in a breast cancer cell line [22]. Thus, lycopene may also prevent or slow down PCa growth through alternating patterns of DNA methylation. Therefore, we evaluated whether lycopene would have an effect on DNA methylation in human PCa cell lines.

## 2. Materials and Methods

### 2.1. Cell Culture and Treatment

Human prostate carcinoma cell lines, androgen-dependent LNCaP cells, and androgen-independent PC-3 cells, obtained from the Shanghai Type Culture Collection (China), were grown in RPMI1640 medium (HyClone, Logan, UT, USA) containing 10% (v/v) fetal bovine serum (HyClone) without the use of antibiotics. Cells were kept in a humidified incubator under standard conditions (5% CO_2_, 95% air at 37°C).

Lycopene, Tetrahydrofurancontaining, butylhydroxytoluene (THF/BHT), 5-Aza-2′-deoxycytidine (AZA), and dimethylsulfoxide (DMSO) were purchased from Sigma-Aldrich (St. Louis, MO, USA). A 4 mM stock solution of lycopene was prepared by dissolving lycopene powder in THF/BHT with minimal exposure to air and light [23]. After 48 h of growth, cultures were changed with fresh RPMI 1640 medium and THF/BHT/FBS-lycopene added to each well, resulting in final lycopene concentrations of 0, 5, 10, 20, or 40 *μ*M. Cells were harvested on treatment days 7 and 14. Cells were treated with 5 *μ*M AZA for 4 days prior to being harvested for a positive control.

### 2.2. Genomic DNA Extraction, Bisulfite Modification, and Bisulfite Sequencing PCR

Genomic DNA samples (1 mg), isolated by the Wizard Genomic DNA Purification Kit (Promega, Madison, WI, USA), were treated with bisulfate using the Methylamp DNA Modification Kit (Epigentek, Brooklyn, NY, USA) to convert unmethylated cytosines to uracils. Bisulfite modified DNA was dissolved in 20 *μ*L water and stored at −80°C. The methylation status for each CpG site was determined by BSP. For BSP, 8 *μ*L of each sample of bisulfite-treated DNA was added to a total reaction volume of 50 *μ*L AmpliTaq Gold PCR Master Mix (Applied Biosystems, Carlsbad, CA, USA). Promoter structure of GSTP1 and sequences for GSTP1, LINE-1, and PC-3 are shown in Supplementary Figures 1 and 2 available online at http://dx.doi.org/10.1155/2014/620165. Primers were designed and synthesized by Sangon Biotech Shanghai Co. (China). Primer sequences for GSTP1 [24], ALU, and LINE-1 [25] are listed in Table 1. Polymerase chain reaction was carried out at 98°C for 4 minutes, followed by 20 cycles of 94°C for 45 seconds, 65°C (−0.5°C each cycle) for 45 seconds, and 72°C for 1 minute. A final incubation at 72°C for 8 minutes was performed. The first-stage PCR product was then amplified using 20 additional cycles of 94°C for 45 seconds, 56°C for 45 seconds, and 72°C for 1 minute. A final incubation at 72°C for 8 minutes was performed. The PCR was repeated for three times and the PCR products were purified using a QIAquick PCR purification kit (Qiagen, Valencia, CA, USA) and cloned in pGEM-T Easy vector system II (Promega Corp., Madison, WI, USA). Randomly selected 5 clones for specific region of each gene were sent to Sangon Biotech (Shanghai, CHN) for sequencing.

### 2.3. RNA Isolation and Quantitative Real-Time Polymerase Chain Reaction

Total RNA was extracted from cell samples by using Trizol (Invitrogen, Carlsbad, CA, USA). Total RNA (1 *μ*g) from each sample was treated with DNase and converted to complementary DNA (cDNA) using a PrimeScript RT Reagent kit (Takara, Dalian, China). A Stratagene Mx3000p Multiplex Quantitative PCR System with SYBR Premix DimerEraser (Takara) was used for the evaluation of mRNA levels.

Quantitative polymerase chain reaction (qPCR) was carried out at 95°C for 2 minutes, followed by 30 cycles of 94°C for 30 s, 60°C for 30 s, and 72°C for 30 s. Reactions were performed in triplicate and products were subjected to melting curve analysis and visualized on an agarose gel to confirm size. Relative gene expression levels were calculated using the 2^−ΔΔCt^ method [26]. Mean expression levels were represented as the ratio between the different detectors and ACTB expression. The primers for GSTP1 [27] and ACTB are listed in Table 2.

### 2.4. Western Blotting

Cells were lysed with RIPA buffer containing proteinase inhibitors then centrifuged at 12,000 g for 30 min at 4°C. The total protein was quantified and separated by 10% sodium dodecyl sulfate-polyacrylamide gel electrophoresis (SDS-PAGE) and transferred to a polyvinylidene fluoride membrane (Millipore, Billerica, MA, USA). After blocking with 5% nonfat milk in Tris-buffered saline with Tween 20 (pH7.4) for 1 h, the membrane was incubated at 4°C overnight with primary antibodies to GSTP1 (Abcam, Cambridge, MA, USA) and *β*-actin (Sigma-Aldrich) and subsequently incubated with appropriate horseradish peroxidase-conjugated secondary antibody followed by detection using an enhanced chemiluminescence kit (Beyotime, Shanghai, China). Densitometry measurements were performed using Quantity One v4.4.0 (Bio-Rad Laboratories, Hercules, CA, USA). The intensity of each band was normalized using *β*-actin.

### 2.5. Immunofluorescence

Cells were seeded onto six-pore plates and covered by a collagen I (Sigma-Aldrich) pretreated cover glass. Each group was subjected to its own designated treatment regimen, and the cells were cultured at 37°C for 24, 48, and 72 hours, respectively. Cells were fixed with 4% paraformaldehyde (Sigma-Aldrich) at 37°C for 10 minutes. The fixed cells were then permeabilized with 0.1% Triton X-100 (Sigma-Aldrich) for 5 minutes at room temperature. Cells were incubated overnight at 4°C in primary rabbit polyclonal antibodies specific for GSTP1 (Abcam, Cambridge, MA, USA) (1 : 200 dilution). Cells were incubated away from light for one hour at room temperature in 1 : 1000 Cy3 mouse anti-rabbit IgG secondary antibody (Santa Cruz, CA, USA). Nuclei were counterstained with 4′,6-diamidino-2-phenylindole (DAPI) (Biyuntian, Jiangsu, China). Coverslips were sealed with sealing liquid (Beyotime). Photomicrographs were taken with an Olympus DP70 (Olympus, Tokyo, Japan). Ten visual fields were observed at random in each coverslip.

### 2.6. Statistical Analysis

SPSS v 11.5 (SPSS, Inc., Chicago, IL, USA) was used for the statistical analyses. Data are presented as the mean ± S.D. Differences between the lycopene-treated cells and control cells were analyzed using Student's* t-*test, Wilcoxon rank sum test, or 2-sided Fisher exact test. A *P* value of <0.05 was considered significant.

## 3. Results

### 3.1. Lycopene Treatment Induced Significant Demethylation of the GSTP1 Promoter in a Human Androgen-Independent Prostate Cancer Derived PC-3 Cell Line

**Figure 1:**
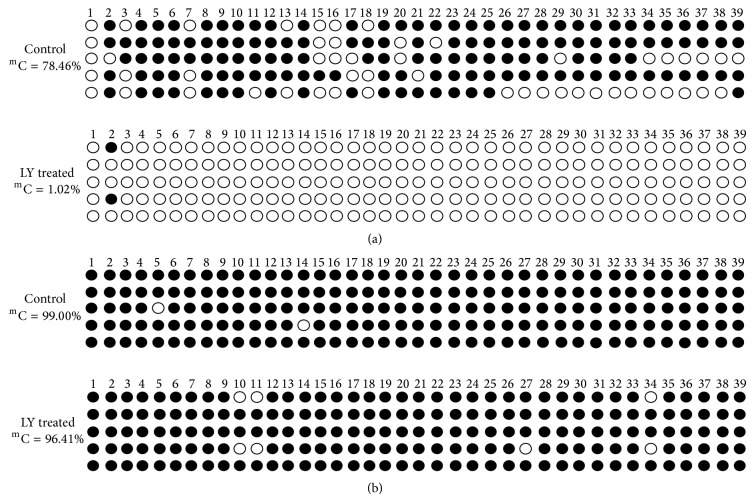
Effects of lycopene treatment on DNA methylation of GSTP1 gene in PCa cells. (a) methylation levels of the GSTP1 promoter in lycopene treated PC-3 and control cells. (b) methylated cytosines of GSTP1 promoter in lycopene treated LNCaP cells and control cells. ^m^C, methylated cytosine. LY, lycopene.

To determine whether lycopene treatment could reverse DNA methylation on GSTP1 CpG island, we used BSP to quantitatively assess the methylation status of each CpG site in the flanking regions of GSTP1 gene. Five clones were randomly selected and used for BSP analyses. There were 39 CpG sites spanning the promoter and exon 1 region on GSTP1. The CpG sites in GSTP1 were 99.00% methylated in LNCaP and 78.46% methylated in PC-3 cell lines, indicating that the sequenced region in the two prostate cancer cell lines was hypermethylated at baseline. Following 14 days of 10 *μ*M lycopene treatment, methylation of the GSTP1 promoter region was significantly reduced in PC-3 cells (1.02%) compared to controls (*P* < 0.05) (Figure 1(a)), but no effects on the promoter of GSTP1 in the LNCaP cell line (96.41%) were noted (*P* > 0.05) (Figure 1(b)).

### 3.2. Lycopene Treatment Increased the mRNA and Protein Levels of GSTP1 in PC-3 Cells but Not in LNCaP Cells

**Figure 2:**
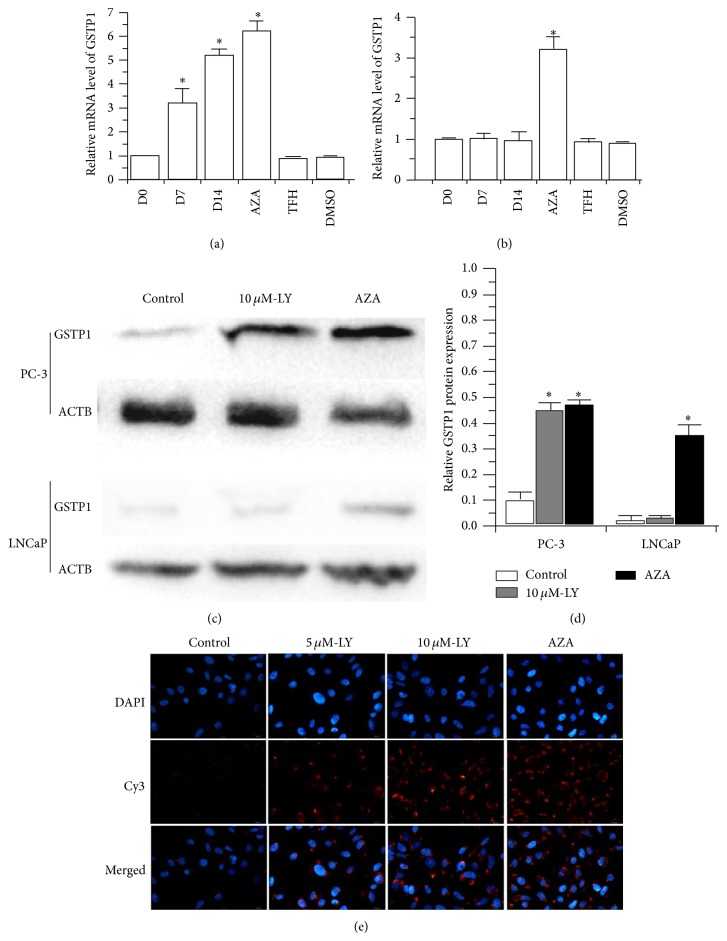
Lycopene induced expression of GSTP1 in PCa cell lines. qPCR detection of the relative mRNA level of GSTP1 in PC-3 (a) and LNCaP (b) cells with 10 *μ*M of lycopene at 0 days (D0), 7 days (D7), and 14 days (D14) and treated with the DNMT inhibitor AZA and vehicles DMSO and TFH. Western blot analysis of GSTP1 protein in PC-3 (c) and LNCaP (d) cells with different treatments. (e) Immunofluorescence detection of GSTP1 protein expression in PC-3 cells treated by lycopene and AZA. LY, lycopene.

We evaluated whether demethylation of the GSTP1 promoter by lycopene treatment on PCa cells would reactivate GSTP1 mRNA expression. As expected, lycopene significantly upregulated the mRNA level of GSTP1 in a time-dependent manner over vehicle control. Following 7 days of treatment with 10 *μ*M of lycopene, PC-3 cells produced approximately half as much GST1 as observed in the positive control cells treated by AZA, a DNMT inhibitor (Figure 2(a)). This indicated that GSTP1 gene expression could be induced by lycopene treatment. However, in the LNCaP cells, 10 *μ*M lycopene treatment did not increase GSTP1 mRNA levels (*P* > 0.05) (Figure 2(b)).

Western blotting was then used to detect if protein expression of GSTP1 was affected by lycopene exposure. GSTP1 protein expression was significantly increased by 10 *μ*M lycopene and AZA treatment in PC-3 cells. However, lycopene did not increase the expression of GSTP1 in LNCaP cells (Figures 2(c) and 2(d)). After 14 days of treatment with lycopene at concentration of 10 *μ*M, upregulated GSTP1 protein expression was observed in PC-3 cells using immunofluorescence (Figure 2(e)). These results further validate the effect of lycopene on the demethylation of GSTP1 gene.

### 3.3. Lycopene Treatment Decreased Protein Level of DNMT3A in PC-3 Cell Line

**Figure 3:**
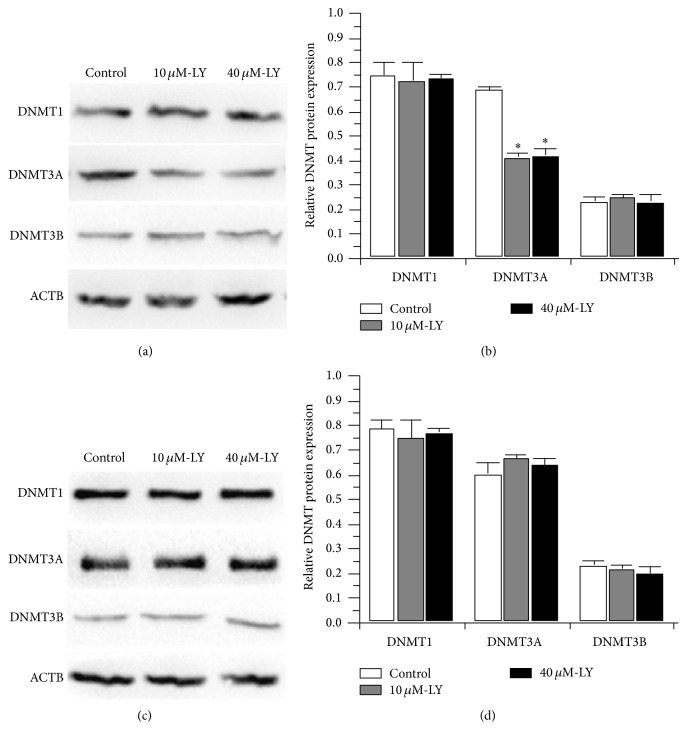
Western blot analysis protein expression of DNMT1, DNMT3A, and DNMT3B in PC-3 ((a) and (b)) and LNCaP cell lines ((c) and (d)) induced by 10 *μ*M and 40 *μ*M lycopene. LY, lycopene.

Three active DNA methyltransferases (DNA MTase), named DNMT1, DNMT3A, and DNMT3B, have been identified in mammals and may have roles in the development of PCa [28]. Although active DNA demethylation by replication-independent processes is known to occur in mammals, the enzymes responsible for it have not been conclusively identified. DNA demethylation can be achieved passively through the failure of maintenance methylation after DNA replication, especially following the repression of DNMTs [29]. Western blotting was used to confirm the effect of the lycopene on protein expression of DNMT1, DNMT3A, and DNMT3B in PC-3 and LNCaP cell lines. In lycopene treated PC-3 cells, expression of DNMT3A was repressed significantly (*P* < 0.01). In contrast, no change was detected in DNMT1 and DNMT3B (both *P* > 0.05; Figure 3). This indicated that DNMT3A may be involved in the passive demethylation of GSTP1.

### 3.4. Lycopene Treatment Did Not Induce Demethylation of LINE-1 and ALU in the Human PC-3 Cell Line

**Figure 4:**
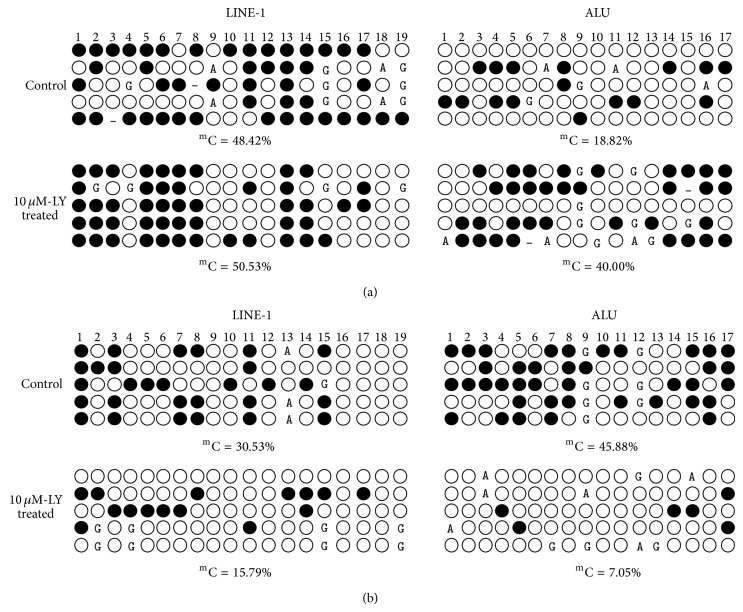
Effects of lycopene treatment on DNA methylation of LINE-1 and ALU elements in PCa cells. (a) Methylated cytosine in LINE-1 and ALU of lycopene treated PC-3 and control cells. (b) Methylated cytosine in LINE-1 and ALU of lycopene treated LNCaP and control cells. Each row of circles represents a single cloned allele (five clones per cell line). Filled and open circles indicate methylated and unmethylated cytosines, respectively.

CpG sites located within LINE-1 and ALU elements are normally heavily methylated in normal human tissues, and a loss of DNA methylation of these elements is closely associated with cancer progression [30]. Therefore, we analyzed the effect of lycopene on the methylation status of LINE-1 and ALU in androgen-dependent LNCaP and androgen-independent PC-3 cell lines. After 14 days of 10 *μ*M lycopene treatment, demethylation of both LINE-1 and ALU elements was not induced in PC-3 cells. The methylation level for LINE-1 was 48.42% and 50.53% in lycopene treated cells and the control, respectively. Lycopene treated PC-3 cells showed 40.00% methylation of CpGs in ALU compared to 18.82% in control cells (Figure 4(a)). These indicated that lycopene would not lead to hypomethylation of LINE-1 and ALU in androgen-independent PC-3 cell lines.

In contrast to the PC-3 cells, 14 days treatment with 10 *μ*M lycopene significantly decreased methylation levels of LINE-1 elements in LNCaP cells, with the decrease in methylated cytosine from 30.53% to 15.79% (*P* < 0.05). Similar effects were noted with the methylation status of ALU; the percentage of methylated cytosine in ALU was downregulated from 45.88% to 7.05% (*P* < 0.05) (Figure 4(b)).

## 4. Discussion

Chemotherapy is the typical treatment for patients with advanced stage of PCa; however, it is largely ineffective. Aberrant DNA methylation of GSTP1 gene associated with inappropriate gene-silencing is an important carcinogenic event in prostate adenocarcinoma [4]. Unlike genetic modifications, epigenetic modification by DNA methylation is reversible, making it a promising new target for therapeutics. Recently, evidence for a potential role of CpG island methylation on drug responses of GSTP1 has grown.

Chemopreventive agents with mechanisms proposed to target DNA methylation include many bioactive food components such as dietary polyphenols, genistein, polyphenols, (−)-epigallocatechin-3-gallate (EGCG), curcumin, and lycopene [31]. Lycopene treatment has been shown to have a potential role in demethylation, as it has been shown to partially demethylate the GSTP1 promoter and restore the GSTP1 expression in breast cancer cells [22]. In this study, we detected heterogeneous methylation of GSTP1 5′ CpG islands in androgen-independent PC-3 and androgen-dependent LNCaP cell lines using a bisulfite sequencing PCR method. This suggested that CpG islands within the promoter region of the GSTP1 were methylated, leading to transcriptional silencing of the GSTP1. A treatment of 10 *μ*M lycopene was used in the present study. Similar to results observed by Liu and Erdman Jr. [32], lycopene did not induce demethylation on the CpG islands of GSTP1 promoter or increase GSTP1 expression in the androgen-dependent LNCaP cell line. However, in contrast with the results observed in LNCaP cells, the same treatment specifically demethylated the GSTP1 promoter and induced mRNA and protein expression of GSTP1 in androgen-independent PC-3 cells. These data suggested that the demethylation effect of lycopene might be androgen-independent.

DNA is methylated by DNA methyltransferases, a family of enzymes that comprises maintenance methyltransferase DNMT1 and the maintenance methyltransferases DNMT3A and DNMT3B in human cells. A lack of methylation of hemimethylated sites by DNMT1 or a failure of DNMT3A or DNMT3B to retain dense methylation of a normally highly methylated region may lead to passive demethylation [33–35]. In the present study, the GSTP1 promoter was demethylated, and lycopene induction reactivated gene expression in androgen-independent PC-3 cells. Furthermore, DNMT3A, but not DNMT1 and DNMT3B, was significantly repressed in lycopene treated PC-3 cells. This result indicated that the downregulation of DNMT3A might be involved in lycopene-induced promoter demethylation or was sufficient to remain dense methylation of GSTP1. However, it is still unclear whether the lycopene affects DNMT3A by interacting with the catalytic site of the DNMT3A molecule.

The reexpression of methylation-inactivated tumor suppressor genes by DNMT inhibitors has provided an effective approach in cancer prevention and therapy. In recent years, the DNMT inhibitory properties of some bioactive food components have been shown to have cancer inhibitory activities through demethylating methylation-silenced cancer-causing genes [28]. However, DNMT inhibitors have also been shown to induce or promote tumorigenesis via DNA hypomethylation at various genomic sequences, particularly the short and long interspersed repeat elements. DNA hypomethylation of those sequences can result in increased genomic instability and has been associated with human cancer [36].

LINE-1 stands for long interspersed nucleotide elements and makes up at least 18% of the human genome. ALU repetitive elements represent one of the most abundant short interspersed nucleotide elements, contributing almost to 11% of the human entire genome. LINE-1 and ALU elements normally undergo heavy methylation in normal human tissues, and a loss of DNA methylation of these elements is closely associated with cancer progression [30]. Thus, alteration in the methylation status of LINE-1 and ALU elements is considered to be a useful surrogate marker for global DNA methylation content. In the present study, we evaluated the effects of lycopene on these repeat elements in PC-3 and LNCaP cell lines. These results showed hypomethylation of ALU and LINE-1 elements in two PCa cell lines. Further analysis showed that global LINE-1 methylation levels in lycopene-treated androgen-dependent LNCaP cells were significantly lower compared with the control cells. The same effects of lycopene treatment on the methylation of ALU elements were also observed in LNCaP cells. However, lycopene treatment did not induce DNA demethylation of LINE-1 and ALU elements in an androgen-independent prostate cancer PC-3 cell line. These results indicated that the potential protective effects of lycopene on prostate might be specific to androgen-independence.

In the present study, we focus on the effects of lycopene on DNA methylation modulation in vitro, which will offer exciting new opportunities for exploring the role of lycopene in influencing the biology of PCa. However, additional in vivo studies are needed to address the effects on methylation modulation through dietary lycopene.

## Supplementary Material

Supplementary Figure 1. Structure of the human GSTP1 gene. An enlarged view of the 423-bp promoter region shows the transcriptional start site (TSS) and 39 CpG sites (purple dashes). Dark green boxes denote CpG islands associated with the 5' untranscriptional region and coding region of GSTP1. Black boxes indicate exons of GSTP1. CpG, cytosine-guanine dinucleotide. Supplementary Figure 2. Bisulfite sequencing of the GSTP1 promoter region, LINE-1 and ALU. Numbers represent CpG sites; Boxes represent locations of sequencing primers

## Figures and Tables

**Table 1:** The list of bisulfite sequencing primers. Y indicates cytosine or thymine; R indicates adenosine or guanine.

Gene	Primer	Product size
M-GSTP1-F	5′-GGATYGTAGYGGTTTTAGGGAAT-3′	423 bp
M-GSTP1-R	5′-CATACTAAAAACTCTAAACCCCATC-3′	
M-ALU-F	5′-GTTTGTAATTTTAGTATTTTGGGAGGT-3′	247 bp
M-ALU-R	5′-TCTATCRCCCAAACTAAAATACAATAAC-3′	
M-LINE1-F	5′-GTTTATTTTATTAGGGAGTGTTAGATAGTG-3′	303 bp
M-LINE1-R	5′-TTAATCTCAAACTACTATACTAACAATCAAC-3′	

**Table 2:** The list of qPCR primers.

Gene	Primer	*T*_*m*_
GSTP1-F	5′-GGGCAGTGCCTTCACATAGT-3′	60°C
GSTP1-R	5′-GGAGACCTCACCCTGTACCA-3′	
ACTB-F	5′-GTTTGTAATTTTAGTATTTTGGGAGGT-3′	60°C
ACTB-R	5′-TTAATCTCAAACTACTATACTAACAATCAAC-3′	

## References

[1] Jemal A., Siegel R., Xu J., Ward E. (2010). Cancer statistics, 2010. *CA Cancer Journal for Clinicians*.

[2] Nelson W. G., de Marzo A. M., Yegnasubramanian S. (2009). Epigenetic alterations in human prostate cancers. *Endocrinology*.

[3] Lee W.-H., Morton R. A., Epstein J. I., Brooks J. D., Campbell P. A., Bova G. S., Hsieh W.-S., Isaacs W. B., Nelson W. G. (1994). Cytidine methylation of regulatory sequences near the π-class glutathione S-transferase gene accompanies human prostatic carcinogenesis. *Proceedings of the National Academy of Sciences of the United States of America*.

[4] Lin X., Tascilar M., Lee W.-H., Vles W. J., Lee B. H., Veeraswamy R., Asgari K., Freije D., Van Rees B., Gage W. R., Bova G. S., Isaacs W. B., Brooks J. D., Deweese T. L., De Marzo A. M., Nelson W. G. (2001). GSTP1 CpG island hypermethylation is responsible for the absence of GSTP1 expression in human prostate cancer cells. *The American Journal of Pathology*.

[5] Wu T., Giovannucci E., Welge J., Mallick P., Tang W.-Y., Ho S.-M. (2011). Measurement of GSTP1 promoter methylation in body fluids may complement PSA screening: a meta-analysis. *British Journal of Cancer*.

[6] Brooks J. D., Weinstein M., Lin X., Sun Y., Pin S. S., Bova G. S., Epstein J. I., Isaacs W. B., Nelson W. G. (1998). CG island methylation changes near the GSTP1 gene in prostatic intraepithelial neoplasia. *Cancer Epidemiology Biomarkers and Prevention*.

[7] Nakayama M., Bennett C. J., Hicks J. L., Epstein J. I., Platz E. A., Nelson W. G., De Marzo A. M. (2003). Hypermethylation of the human glutathione S-transferase-*π* gene (GSTP1) CpG island is present in a subset of proliferative inflammatory atrophy lesions but not in normal or hyperplastic epithelium of the prostate: a detailed study using laser-capture microdissection. *American Journal of Pathology*.

[8] Meiers I., Shanks J. H., Bostwick D. G. (2007). Glutathione S-transferase pi (GSTP1) hypermethylation in prostate cancer: review 2007. *Pathology*.

[9] Nelson C. P., Kidd L. C. R., Sauvageot J., Isaacs W. B., De Marzo A. M., Groopman J. D., Nelson W. G., Kensler T. W. (2001). Protection against 2-hydroxyamino-1-methyl-6-phenylimidazo[4,5-b]pyridine cytotoxicity and DNA adduct formation in human prostate by Glutathione S-Transferase P1. *Cancer Research*.

[10] Henderson C. J., Smith A. G., Ure J. (1998). Increased skin tumorigenesis in mice lacking pi class glutathione S-transferases. *Proceedings of the National Academy of Sciences of the United States of America*.

[11] Yoo C. B., Cheng J. C., Jones P. A. (2004). Zebularine: a new drug for epigenetic therapy. *Biochemical Society Transactions*.

[12] Davis C. D., Uthus E. O. (2004). DNA methylation, cancer susceptibility, and nutrient interactions. *Experimental Biology and Medicine*.

[13] Nandakumar V., Vaid M., Katiyar S. K. (2011). (-)-Epigallocatechin-3-gallate reactivates silenced tumor suppressor genes, Cip1/p21 and p16INK4a, by reducing DNA methylation and increasing histones acetylation in human skin cancer cells. *Carcinogenesis*.

[14] Fu S., Kurzrock R. (2010). Development of curcumin as an epigenetic agent. *Cancer*.

[15] Pandey M., Shukla S., Gupta S. (2010). Promoter demethylation and chromatin remodeling by green tea polyphenols leads to re-expression of GSTP1 in human prostate cancer cells. *International Journal of Cancer*.

[16] Najm W., Lie D. (2008). Dietary supplements commonly used for prevention. *Primary Care - Clinics in Office Practice*.

[17] Ansari M. S., Sgupta N. P. (2005). A comparison of lycopene and orchidectomy vs orchidectomy alone in the management of advanced prostate cancer. *BJU International*.

[18] Ansari M. S., Ansari S. (2005). Lycopene and prostate cancer. *Future Oncology*.

[19] Lu Q.-Y., Hung J.-C., Heber D., Go V. L. W., Reuter V. E., Cordon-Cardo C., Scher H. I., Marshall J. R., Zhang Z.-F. (2001). Inverse associations between plasma lycopene and other carotenoids and prostate cancer. *Cancer Epidemiology Biomarkers and Prevention*.

[20] Gann P. H., Ma J., Giovannucci E., Willett W., Sacks F. M., Hennekens C. H., Stampfer M. J. (1999). Lower prostate cancer risk in men with elevated plasma lycopene levels: results of a prospective analysis. *Cancer Research*.

[21] Kucuk O., Sarkar F. H., Djuric Z., Sakr W., Pollak M. N., Khachik F., Banerjee M., Bertram J. S., Wood D. P. (2002). Effects of lycopene supplementation in patients with localized prostate cancer. *Experimental Biology and Medicine*.

[22] King-Batoon A., Leszczynska J. M., Klein C. B. (2008). Modulation of gene methylation by genistein or lycopene in breast cancer cells. *Environmental and Molecular Mutagenesis*.

[23] Lin C.-Y., Huang C.-S., Hu M.-L. (2007). The use of fetal bovine serum as delivery vehicle to improve the uptake and stability of lycopene in cell culture studies. *The British Journal of Nutrition*.

[24] Gonzalgo M. L., Pavlovich C. P., Lee S. M., Nelson W. G. (2003). Prostate cancer detection by GSTP1 methylation analysis of postbiopsy urine specimens. *Clinical Cancer Research*.

[25] Yang A. S., Estécio M. R. H., Doshi K., Kondo Y., Tajara E. H., Issa J.-P. J. (2004). A simple method for estimating global DNA methylation using bisulfite PCR of repetitive DNA elements. *Nucleic Acids Research*.

[26] Livak K. J., Schmittgen T. D. (2001). Analysis of relative gene expression data using real-time quantitative PCR and the 2-ΔΔCT method. *Methods*.

[27] Bakker J., Lin X., Nelson W. G. (2002). Methyl-CpG binding domain protein 2 represses transcription from hypermethylated π-class glutathione S-transferase gene promoters in hepatocellular carcinoma cells. *Journal of Biological Chemistry*.

[28] Li Y., Tollefsbol T. O. (2010). Impact on DNA methylation in cancer prevention and therapy by bioactive dietary components. *Current Medicinal Chemistry*.

[29] Chen Z. X., Riggs A. D. (2011). DNA methylation and demethylation in mammal. *Journal of Biological Chemistry*.

[30] Cho N.-Y., Kim B.-H., Choi M., Yoo E. J., Moon K. C., Cho Y.-M., Kim D., Kang G. H. (2007). Hypermethylation of CpG island loci and hypomethylation of LINE-1 and Alu repeats in prostate adenocarcinoma and their relationship to clinicopathological features. *Journal of Pathology*.

[31] Huang J., Plass C., Gerhäuser C. (2011). Cancer chemoprevention by targeting the epigenome. *Current Drug Targets*.

[32] Liu A. G., Erdman J. W. (2011). Lycopene and apo-10′-lycopenal do not alter DNA methylation of GSTP1 in LNCaP cells. *Biochemical and Biophysical Research Communications*.

[33] Ficz G., Hore T. A., Santos F., Lee H. J., Dean W., Arand J., Krueger F., Oxley D., Paul Y.-L., Walter J., Cook S. J., Andrews S., Branco M. R., Reik W. (2013). FGF signaling inhibition in ESCs drives rapid genome-wide demethylation to the epigenetic ground state of pluripotency. *Cell Stem Cell*.

[34] Kagiwada S., Kurimoto K., Hirota T., Yamaji M., Saitou M. (2013). Replication-coupled passive DNA demethylation for the erasure of genome imprints in mice. *The EMBO Journal*.

[35] Ohno R., Nakayama M., Naruse C., Okashita N., Takano O., Tachibana M., Asano M., Saitou M., Seki Y. (2013). A replication-dependent passive mechanism modulates DNA demethylation in mouse primordial germ cells. *Development*.

[36] Wu H.-C., Delgado-Cruzata L., Flom J. D., Perrin M., Liao Y., Ferris J. S., Santella R. M., Terry M. B. (2012). Repetitive element DNA methylation levels in white blood cell DNA from sisters discordant for breast cancer from the New York site of the breast cancer family registry. *Carcinogenesis*.

